# Supplementary material for: Assessment of knowledge and perception of prescribers towards rational medicine use in the Ashanti Region of Ghana
Source: PLoS One. 2024 Oct 31;19(10):e0308406. doi: 10.1371/journal.pone.0308406 (PMC11527319; doi:10.1371/journal.pone.0308406)
Supplement: S1 Tool — (DOCX) [file pone.0308406.s001.docx]

**S1 Tool**

1. What is your sex?
   1. Male
   2. Female
2. What is your age (in years as of your last birthday) ……………?
3. What is your marital status?

a. Married

b. Single

c. Divorced

d. Widowed

1. What is the classification of your hospital?

a. Primary Hospital

b. Regional Hospital

1. What is the ownership type of your hospital?

a. Government

b. Private

c. Christian Mission

d. Islamic Mission

1. What is your professional designation as a prescriber? a. Medical doctor

b. Physician assistant

c. Physician assistant intern

d. Medical doctor intern

e. Nurse

f. Other (please specify) ……………………………………

1. What is your employment status in this hospital?

a. Permanent

b. Temporal

c. Trainee

1. How many years have you been working as a prescriber?

a. 1-3 years

b. 4-6 years

c. 7 – 10 years

d. ≥11 years

1. What is the duration of your employment as a prescriber in your current hospital?

a. 1-3 years

b. 4-6 years

c. 7-10 years

d. ≥11 years

1. Are drug bulletins created by the pharmacy department and provided to prescribers in your hospital?

a. Yes

b. No

1. How frequently are the drug bulletins in your hospital updated?

a. Monthly

b. Quarterly

c. Annually

d. Never

1. What sources do you consult when prescribing medicines? Please select all that apply.

a. Standard Treatment Guidelines (STG) of Ghana

b. British National Formulary (BNF)

c. Medscape

d. Drug bulletin from hospital pharmacy

e. Other (Please specify) ……………………………………

1. Is your preferred prescribing reference source available in the hospital?

a. Yes

b. No

1. Which of the following criteria is NOT considered when selecting medications for inclusion in the Essential Medicines List (EML)?
   1. Safety
   2. **Brand recognition**
   3. Efficacy
   4. Cost-effectiveness
2. Among the following, which prescription reference source typically has the least amount of evidence?

a. **Expert opinion**

b. Recommendations from randomized control trials

c. Essential Medicines List of Ghana

d. Recommendations from systematic reviews

1. The generic name of a medicine is also referred to as ……...

a. brand name

b. innovator name

c. **non-proprietary name**

d. trade name

1. By Ministry of Health guidelines, all medicines on a prescription should be written in a. **Generic names**

b. Brand names

c. Innovator names

d. All the above

1. Which of the following features is not required in a valid prescription?

a. Age of patient

b. Sex of patient

c. Name of patient

d. **Signature of patient**

e. Name of the drug

e. Dosage, frequency, and duration of the drug

f. Signature of prescriber

1. Which of the following statements best describes the rational use of injections?
2. Injections should be used for all types of illnesses to ensure rapid treatment.
3. **Injections should only be used when absolutely necessary and when no alternative treatments are available.**
4. Injections should be administered without considering patient preferences or medical history.
5. Injections should be the first-line treatment for all medical conditions.
6. What is the recommended approach for treating uncomplicated malaria according to the Ministry of Health's antimalarial policy?
7. **Starting and completing with oral antimalarial medicines.**
8. Starting with a combination of oral and injectable antimalarial medicines simultaneously.
9. Starting with an injection followed by oral antimalarial medicines.
10. Starting with non-pharmacological interventions before considering any medication.
11. Which of the following is not a Ministry of Health (MOH) guideline on prescribing medicines?
12. **Medicines that are not available in the hospital should not be written in patient medical records.**
13. Prescriptions going out of the hospital should have the name and phone number of the prescriber.
14. All prescriptions should be written in ink and signed.
15. All prescriptions should indicate the diagnosis.
16. Which of the following scenarios would not typically warrant the prescription of antibiotics?

a. Patient A, an adult experiencing a fever caused by malaria

b. Patient B, presents with a fever and runny nose

c. Patient C, exhibiting diarrhoea without fever

d. **All the above**

e. None of the above

1. In the context of rational use of medicines, under what conditions would you prefer to prescribe one medicine over the other?

a. Availability in the hospital pharmacy

b. Patient preference

c. **Evidence of efficacy and safety from reference sources**

d. Personal preference

e. All the above

1. In your view, who should prioritize promoting rational medicine use?
   a. Prescriber
   b. Dispenser
   c. Nurse
   d. **All the above**e. None of the above
2. In the context of rational medicine use, the prescriber's primary concern regarding patient welfare should not only be to achieve a cure but also to address drug use problems that may arise
   1. Strongly agree
   2. Agree
   3. Neither agree or disagree
   4. Disagree
   5. Strongly disagree

Top of Form

1. Irrational medicine use does affect antimicrobial resistance
   1. Strongly agree
   2. Agree
   3. Neither agree or disagree
   4. Disagree
   5. Strongly disagree
2. Injections are no more efficacious than oral medications
   1. Strongly agree
   2. Agree
   3. Neither agree or disagree
   4. Disagree
   5. Strongly disagree
3. A patient who is on too many medicines concurrently can develop adverse drug reactions due to drug-drug interactions
   1. Strongly agree
   2. Agree
   3. Neither agree or disagree
   4. Disagree
   5. Strongly disagree
4. Irrational prescribing of medicines can cause or prolong the hospitalization of a patient
   1. Strongly agree
   2. Agree
   3. Neither agree or disagree
   4. Disagree
   5. Strongly disagree
5. To promote rational medicine usage, medications listed on any formulary should be categorized, and their usage should be restricted based on the hospital's level, experience, and specialty of the prescriber.
   1. Strongly agree
   2. Agree
   3. Neither agree or disagree
   4. Disagree
   5. Strongly disagree
6. Are you aware of the existence of a Drugs and Therapeutics Committee (DTC) in your Hospital? a. Yes

b. No

1. Are you aware of any Rational Use of Medicine (RUM) Survey conducted in your hospital in the past 6 months?

a. Yes

b. No

1. If Yes to Q32 above, do you know about the findings in the survey?

a. Yes

b. No

1. Have you taken part in any Rational Use of Medicines training in the past one year?

a. Yes

b. No

Thank you for your time
