# Supplementary material for: Assessment of knowledge and perception of prescribers towards rational medicine use in the Ashanti Region of Ghana
Source: PLoS One. 2024 Oct 31;19(10):e0308406. doi: 10.1371/journal.pone.0308406 (PMC11527319; doi:10.1371/journal.pone.0308406)
Supplement: Attachment — Submitted filename: Response to Reviewers.docx [file pone.0308406.s003.docx]

Pharmacy Department

Bekwai Municipal Hospital

P. O. Box 40

Bekwai Ashanti

30^th^ April 2024

Academic Editor

PLOS ONE

Subject: Rebuttal to Reviewer Comments on Manuscript Submission PONE-D-23-33862

Dear Mr A Kakkar,

We appreciate the opportunity to respond to the comments provided by the academic editor and reviewers regarding our manuscript titled "Assessment of Knowledge and Perception of Prescribers towards Rational Medicine Use in the Ashanti Region of Ghana." We have carefully considered each point raised and have made revisions accordingly. Below, we provide point-by-point responses to the concerns raised:

1. We have duly noted the diverse range of healthcare professionals involved in the medication prescribing process in Ghana, as outlined in the methods section on page 6. We acknowledge the inclusion of mental health nurses, disease control officers, and community health nurses categorized as “Others” in the data analysis.
2. A copy of the study questionnaire, including the bolded correct answers to the knowledge questions, has been uploaded as per the reviewer's suggestion.
3. The threshold score for determining good knowledge and perception has been clarified to be 70%, with corrections made in the text regarding the number of items used to assess perception and knowledge. Additionally, it has been indicated in the methods section that knowledge scores, when converted to percentages, have been rounded to one decimal place using standard rounding rules. The perception score analysis has been redone using ordinal logistic regression. This has also been indicated in the method section.
4. We have changed the term "reference source available" to “reference source accessible” under the results subsection. This clarification aims to facilitate better understanding and ease of referencing when necessary.
5. The existence of a national Standard Treatment Guideline (STG) developed by the Ministry of Health of Ghana, along with the process of formulary list development by individual hospitals, has been appropriately addressed. We acknowledge the limitation of not verifying respondents' answers regarding the availability of STG in their hospitals and this has been noted in the study limitations statement.
6. The statement regarding the association between functional Drug and Therapeutic Committees (DTCs) and RUM knowledge has been corrected as per the reviewer's suggestion.
7. The discussion section has been revised to address the unexpected finding regarding the relationship between updates to the drug bulletin and prescriber knowledge. We acknowledge the need for further research to understand the underlying factors contributing to this discrepancy.
8. We have revised the discussion to provide context from prior research on knowledge levels among Ghanaian prescribers, particularly comparing doctors and Community Health Officers, as well as findings from similar studies conducted in Pakistan.
9. The discussion has been redone after the ordinal regression analysis aligned with our study's results.
10. A study limitation statement has been included before the conclusion, highlighting the cross-sectional design's limitations and potential biases introduced by self-reported data.

We believe these revisions have strengthened the manuscript and addressed the concerns raised by the academic editor and reviewers. We thank the editorial team for their valuable feedback and consideration of our submission.

Sincerely,

Richard Delali Agbeko Djochie, PhD

Bekwai Municipal Hospital, Bekwai Ashanti, Ghana

Tel: +233244101944

Email: [richarddjochie@gmail.com](mailto:richarddjochie@gmail.com)
