# Supplementary material for: Assessment of knowledge and perception of prescribers towards rational medicine use in the Ashanti Region of Ghana
Source: PLoS One. 2024 Oct 31;19(10):e0308406. doi: 10.1371/journal.pone.0308406 (PMC11527319; doi:10.1371/journal.pone.0308406)
Supplement: Attachment — Submitted filename: Response to Reviewers.docx [file pone.0308406.s004.docx]

Pharmacy Department

Bekwai Municipal Hospital

P. O. Box 40

Bekwai Ashanti

20^th^ July 2024

Academic Editor

PLOS ONE

Subject: Rebuttal to Reviewer Comments on Manuscript Submission PONE-D-23-33862R1

Dear Dr Amponsah,

We appreciate the opportunity to respond to the comments provided by the academic editor and reviewers regarding our manuscript titled "Assessment of Knowledge and Perception of Prescribers towards Rational Medicine Use in the Ashanti Region of Ghana." We have carefully considered each point raised and have made revisions accordingly. Below, we provide point-by-point responses to the issues raised:

1. In the Discussion section, the first sentence has been rewritten to remove the phrase “solid comprehension” and now reads: "Overall, most participants demonstrated adequate knowledge and a positive perception of the concept of RUM."
2. The term "knowledge" has been used consistently throughout the discussion, replacing "comprehension" and other similar words to avoid ambiguity.
3. In the Conclusion, the term “understanding” has been replaced with “knowledge” as suggested by the reviewer.
4. The references list has been checked to ensure it is complete.

We believe these revisions have strengthened the manuscript and addressed the concerns raised by the academic editor and reviewers. We thank the editorial team for their valuable feedback and consideration of our submission.

Sincerely,

Richard Delali Agbeko Djochie, PhD

Bekwai Municipal Hospital, Bekwai Ashanti, Ghana

Tel: +233244101944

Email: [richarddjochie@gmail.com](mailto:richarddjochie@gmail.com)
